# COVID-19 Mobile Apps: A Systematic Review of the Literature

**DOI:** 10.2196/23170

**Published:** 2020-12-09

**Authors:** Haridimos Kondylakis, Dimitrios G Katehakis, Angelina Kouroubali, Fokion Logothetidis, Andreas Triantafyllidis, Ilias Kalamaras, Konstantinos Votis, Dimitrios Tzovaras

**Affiliations:** 1 Computational Biomedicine Laboratory Foundation for Research and Technology - Hellas–Institute of Computer Science Heraklion Greece; 2 Center for eHealth Applications and Services Foundation for Research and Technology – Hellas, Institute of Computer Science Heraklion Greece; 3 Information Technologies Institute Centre for Research and Technology - Hellas Thessaloniki Greece

**Keywords:** mobile apps, systematic survey, COVID-19, mobile health, eHealth

## Abstract

**Background:**

A vast amount of mobile apps have been developed during the past few months in an attempt to “flatten the curve” of the increasing number of COVID-19 cases.

**Objective:**

This systematic review aims to shed light into studies found in the scientific literature that have used and evaluated mobile apps for the prevention, management, treatment, or follow-up of COVID-19.

**Methods:**

We searched the bibliographic databases Global Literature on Coronavirus Disease, PubMed, and Scopus to identify papers focusing on mobile apps for COVID-19 that show evidence of their real-life use and have been developed involving clinical professionals in their design or validation.

**Results:**

Mobile apps have been implemented for training, information sharing, risk assessment, self-management of symptoms, contact tracing, home monitoring, and decision making, rapidly offering effective and usable tools for managing the COVID-19 pandemic.

**Conclusions:**

Mobile apps are considered to be a valuable tool for citizens, health professionals, and decision makers in facing critical challenges imposed by the pandemic, such as reducing the burden on hospitals, providing access to credible information, tracking the symptoms and mental health of individuals, and discovering new predictors.

## Introduction

The COVID-19 outbreak, which first emerged in China, has spread worldwide. On March 11, 2020, the World Health Organization (WHO) declared COVID-19 as a pandemic [1]. The disease has disrupted global trade, employment, and travel, and many governments had to take strict measures to control the spread of the virus and minimize the burden of morbidity and mortality so that health care systems remain functional [2]. In many countries around the world, citizens have been recommended to stay at home and practice social distancing for as long as possible as a primary measure of preventing the spread of COVID-19.

Although mobile apps are successfully used for managing chronic diseases [3,4], the ongoing COVID-19 pandemic has pushed the need for mobile app solutions at the forefront to reduce the risk of cross-contamination caused by close contact [5-7]. Mobile technology has been leveraged in a number of ways to control the spread of COVID-19. Mobile apps are accessible, acceptable, and easily adopted, and have the ability to support social distancing efforts. As such, they have been widely developed and implemented during the previous months in an attempt to “flatten the curve” of the increasing number of COVID-19 cases, providing knowledge and information to civilians while attempting to relieve the pressure from health care systems.

Despite increasing reliance on mobile health (mHealth) solutions as part of COVID-19–related response plans, major knowledge gaps exist about their utility and efficacy during the current pandemic for both health professionals as well as for the general population. To this direction, this systematic review aims to shed light into studies found in the scientific literature on the use and evaluation of mobile apps for the prevention, management, treatment, or follow-up of COVID-19.

Other recent reviews have focused merely on the exploration of COVID-19 mobile apps in app stores in general [8] or were restricted to apps deployed in specified countries such as the United States, the United Kingdom, and India [9]. Although there are already related generic COVID-19 information and communication technology surveys [10,11], they focus on specific topics such as contact tracing [12,13]; specialized health sectors like pediatric health care delivery [14], mental health [15], epilepsy [16], and palliative care [17]; or countries like India [14], China [18], and the United Kingdom [19]. To the best of our knowledge, there has been no other work dedicated to the systematic review of pragmatic studies that have demonstrated the real-life use and evaluation of COVID-19 mobile apps.

## Methods

### Search Strategy

The bibliographic databases of PubMed and Scopus, along with the global research database on COVID-19 developed by the WHO [20], were searched to identify mHealth apps used for the purposes of prevention, treatment, or management of COVID-19 and assessed in pragmatic studies.

### Eligibility Criteria

In this context, the inclusion criteria for study selection were the following: features of the COVID-19 mobile app should be described, the study should show evidence of the implementation of the COVID-19 mobile app in real life and provide quantitative outcomes, the study should show that clinical professionals were involved in the design or validation of the mobile app, the paper describing the study must have been written in English. Case reports, letters to editors, preprint papers, qualitative studies, surveys or reviews, simulation studies, and studies describing protocols were excluded from the review.

### Study Selection

The string “(mobile health) OR (mhealth) OR (smartphone) OR (mobile phone) OR (mobile application) OR (mobile app) OR (app) AND (COVID-19),” was used for a search within the title, abstract, and keywords of the manuscripts. Authors HK, AT, AK, FL, DGK, and IK independently screened the identified papers to minimize possible errors and bias in the selection process. Any disagreements were resolved by discussion between the authors to reach consensus. The authors first screened the abstracts of the candidate papers for inclusion and assessed their eligibility according to the defined inclusion and exclusion criteria. Moreover, the authors selected the final papers for inclusion after reading the full manuscripts of the eligible papers, as well as their references.

The Effective Public Health Practice Project (EPHPP) tool was adopted to assess the methodological quality of the included studies. The EPHPP tool is suitable for evaluating quantitative studies in a wide range of health-related topics, and it has demonstrated reliability [21]. The included studies were synthesized (by HK, AK, FL, and DGK) according to their target, mobile app main features, study design, number of enrolled participants and their age, follow-up duration, outcomes and whether these were positive or negative, and implications for clinical practice. This systematic review was conducted following the PRISMA (Preferred Reporting Items for Systematic Reviews and Meta-Analyses) guidelines [22]. A completed PRISMA checklist is shown in Multimedia Appendix 1.

## Results

### Statistical Analysis

**Figure 1:**
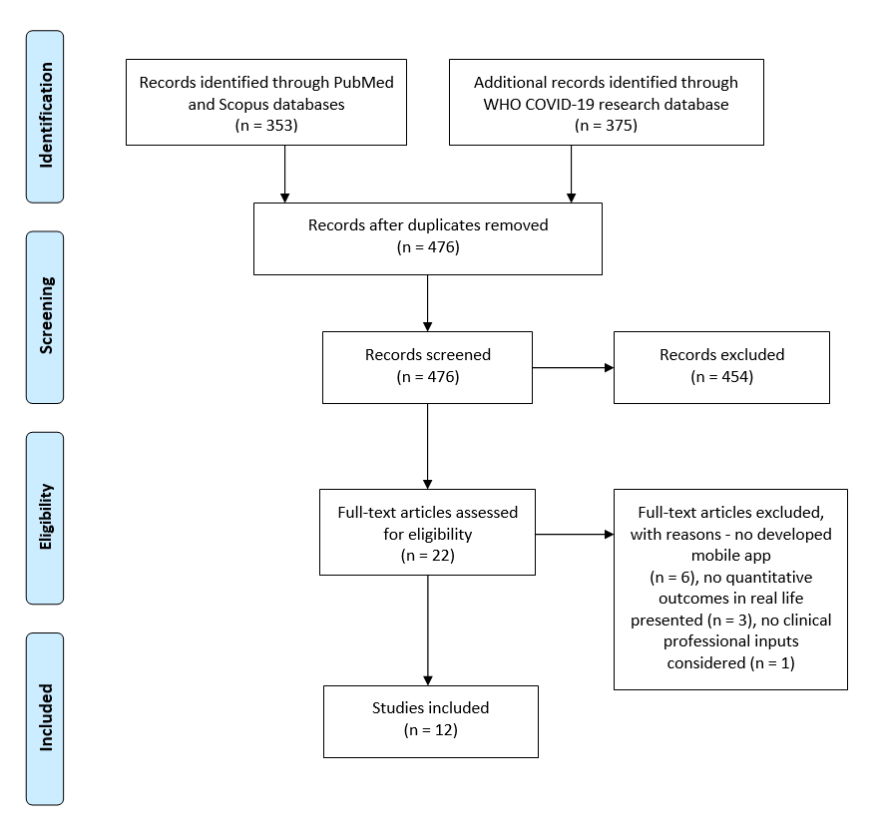
The PRISMA (Preferred Reporting Items for Systematic Reviews and Meta-Analyses) flow diagram. WHO: World Health Organization.

Our last search in June 2020 returned 165 manuscripts from the PubMed database, 188 manuscripts from the Scopus database, and another 375 manuscripts from the WHO research database. All the retrieved records were imported in the Zotero bibliography management software (Center for History and New Media at George Mason University), which identified 252 duplicates. We screened the abstracts of the remaining 476 papers according to our inclusion and exclusion criteria, and 22 papers were found to be eligible. After reading the full text of the papers, the authors agreed to include 12 papers. The screening procedure along with reasons for excluding papers are shown in the PRISMA flow diagram in Figure 1.

### Study Outcomes and Quality Assessment

On the basis of the EPHPP criteria for selection bias, design, confounders, blinding, data collection, and dropouts, we found the methodological quality to be moderate for 2 of the 12 (17%) studies [23,24] and weak for the remaining 10 (83%) studies (Table 1). Most studies were poorly rated because of their observational or cross-sectional nature, insufficient care in controlling for confounders, insufficient reporting on the validity and reliability of the tools used for data collection, and the absence of description on withdrawals and dropouts. The design of a randomized or controlled clinical trial was not reported in any of the reviewed studies.

**Table 1:** Quality assessment of included studies based on the EPHPP criteria.

Study	EPHPP^a^ criteria						Global rating
	SB^b^	SD^c^	CF^d^	BL^e^	DC^f^	WD^g^	
Bae et al [25]	W^h^	W	W	M^i^	W	W	W
Bourdon et al [23]	M	M	M	M	M	W	M
Drew et al [26]	W	W	W	M	W	W	W
Ben Hassen et al [27]	W	W	W	M	S^j^	W	W
Huckins et al [24]	W	M	M	M	M	S	M
Kodali et al [28]	W	W	W	M	W	W	W
Medina et al [29]	M	W	W	M	W	W	W
Menni et al [30]	W	W	S	M	W	W	W
Ros and Neuwirth [31]	M	W	W	M	W	W	W
Timmers et al [32]	M	M	W	M	W	W	W
Yamamoto et al [33]	M	W	W	M	W	W	W
Zamberg et al [34]	W	W	W	M	W	W	W

^a^EPHPP: Effective Public Health Practice Project.

^b^SB: selection bias.

^c^SD: study design.

^d^CF: confounders.

^e^BL: blinding.

^f^DC: data collection methods.

^g^WD: withdrawals and dropouts.

^h^W: weak.

^i^M: moderate.

^j^S: strong.

### Comparison

In the following sections, we provide a comparison of the included studies, in terms of intervention target and main features, study design, and outcome and clinical implications.

#### Intervention Target and Main Features

The reviewed papers varied in intervention target and main features (Table 2). One paper discussed a COVID-19 tracking app informing users about risk assessment and offering relevant advice [28]. The paper did not provide further details about app features. Apps that offered education and information material were discussed in 3 papers. They either targeted patients or health care professionals. Patient education included hospital information (appointments, visiting hours, self-isolation, and general COVID-19 information [32]) and education related to SARS-CoV-2. For health care professionals, they included information about how to handle patients with COVID-19 [31,34]. One paper [25] described how the hospital information system was adapted to handle COVID-19 with the incorporation of relevant templates. An app for recording general health information was discussed in 1 paper [33], and apps for self-assessment focusing on symptoms were found in 1 paper [32]. Self-monitoring based on a symptom diary and health observation data were also reported in 4 papers [26,29,32,33].

**Table 2:** A comparison of intervention target and main features.

Studies	Intervention	Main features
Kodali et al [28]	Tracking app	Risk assessment and advice
Timmers et al [32]	Education and information	Information for patients
Ros and Neuwirth [31] and Zamberg et al [34]	Education and information	Information for health care professionals
Bourdon et al [23]	Hospital information system	Adaptation to pandemic
Yamamoto et al [33]	Health assessment	General
Bae et al [25], Medina et al [29], Timmers et al [32], and Yamamoto et al [33]	Health assessment	Self-monitoring
Medina et al [29]	Home monitoring	Telephone outreach
Bourdon et al [23]	Home monitoring	Vital signs with smart devices
Drew et al [26]	Home monitoring	Internet of Things
Timmers et al [32]	Interactive map	Demographics and health status
Yamamoto et al [33]	Data sharing	Patient email to specific recipients
Medina et al [29]	Data sharing	Nurse outreach
Ben Hassen et al [27]	Student mental health	Behavior during the pandemic
Menni et al [30]	Prediction model	Progress of disease
Huckins et al [24]	Teleophtalmology	Emergency eye care

Home monitoring was handled based on telephone outreach [29], smart vital sign monitoring devices [25], and Internet of Things [27]. An interactive map of user demographics and health status was discussed in 1 paper [32]. Two apps offered data sharing capabilities via email to specific recipients based on patient options [33], or as symptoms were worsening, the app sent messages to be assessed by nursing staff to provide appropriate advice [29]. One paper assessed student behavior to determine whether the mental health of participants changed due to the COVID-19 pandemic [24]. One paper used data collected by Drew et al [26] to develop a prediction model about disease progress as well as tracking the disease progress in real time [30]. Finally, 1 paper [23] reported on a smartphone app to manage emergency eye care.

#### Study Design and Structure of Research

Regarding the *study design*, almost all selected studies were proof of concept, observational studies, or both, aiming to evaluate the practical use, usability, or user satisfaction with the corresponding apps that were developed to support individuals, health care providers, and policy makers during the COVID-19 pandemic (Table 3).

**Table 3:** A comparison of study design and structure of the research.

Study	Study design	Participants, n	Age	Follow-up duration (study period)
Bourdon et al [23]	Online surveys on patient and medical staff satisfaction with the mobile app and the wearables	12 patients and 24 medical staff	Patient mean age: 25 years; no information for medical staff	No follow-up
Huckins et al [24]	Observational cohort study measuring behaviors through the StudentLife smartphone sensing app	500	Mean: 40.7 (SD 20.3; 0.6-92) years. The number of patients older than 60 years was small.	No follow-up
Bae et al [25]	Observational data collection that helped develop predictive models. Participants that were already enrolled in ongoing epidemiologic studies were approached to use this app.	>2 million users; 75% female	Mean: 41 (range: 18-90) years	The launching of the COVID Symptom Study app occurred in the United Kingdom on March 24, 2020, and in the United States on March 29, 2020. 265,851 individuals were enrolled by March 27, 2020.
Drew et al [26]	Observational study on the COVID Symptom Tracker mobile app	5 hospitalized patients and 5 doctors	Patients range: 45-61 years	No follow-up
Ben Hassen et al [27]	The StudentLife app was used for smartphone mobile sensing. Ecological momentary assessments were used to assess depression and anxiety.	217; 67.8% (n=147) were female	Range: 18-22 years at the time of enrollment	178 (82.0%) students provided data during the Winter 2020 term (January 6 to March 13, 2020).
Kodali et al [28]	Observational study using descriptive statistics and thematic analysis on the mHealth^a^ app Arogya Setu.	503 most relevant reviews were identified based on the Google algorithm	Not reported	All reviews that were available publicly and posted in English by the users until April 21, 2020, were included. The start date of app reviews collection was not reported.
Medina et al [29]	Observational cohort study carried out at the Cleveland Clinic, OH, US. It included a self-monitoring app for patient engagement and early intervention.	COVID-19 patients enrolled by May 25, 2020: 1924. Most (85%) patients were enrolled 5 days from symptom onset.	25% (n=483) were older than 60 years, and 3.5% (n=67) were younger than 18 years.	Engagement with MyCare Companion app reached 32%; 25% continued under monitoring for longer than 14 days due to persistent symptoms.
Menni et al [30]	Observational data collection and statistical analysis that helped develop predictive models	Symptoms were reported by 2,450,569 from the UK and 168,293 from the US	Average age for tested positive, tested negative, and not tested: (UK: 41.25, 41.87, and 43.38; US: 41.87, 47.25, and 53.00).	Data analyzed had been collected between March 24 and April 21, 2020.
Ros and Neuwirth [31]	A tutorial feedback survey was conducted. User feedback was requested from health care workers and responders about the presented global public health educational outreach technology.	12,516 users, learners, health care workers, and responders downloaded the app in 1 month.	Not provided	366 replies received during the first 72 h of deploying the survey. During this time period, there were 512 subscribers that had downloaded the app (71.48% response rate).
Timmers et al [32]	Observational cohort study (based on the data collected at the ETZ^b^ hospital), assessed the use of the app as well as its usability. Data were gathered for health care providers and policy makers.	6194 individuals downloaded the app.	Average: 50.87 years	The study focused on data collected between April 1-20, 2020. The app was being used by over 15 hospitals in the Netherlands, Belgium, and Germany, accumulating over 30,000 downloads.
Yamamoto et al [33]	Proof of concept and practical use study in a real-world setting. The study aimed to develop a PHR^c^-based COVID-19 symptom-tracking app to determine whether PHRs could be used for efficient health observation outside a traditional hospital setting. The practical aspects of health observations for COVID-19 using the smartphone or tablet app integrated with PHRs was demonstrated. Moreover, a usability evaluation of the app was carried out based on interviews with help desk managers of the app.	In the context of the active epidemiological investigation period (from March 6-19, 2020) at Wakayama City Public Health Center, 72 individuals who had close contact with a COVID-19 confirmed case were discovered. Among them, 57 had adopted the use of the health observation app.	N/A^d^	The active epidemiological investigation period was carried out from March 6-19, 2020, at Wakayama City Public Health Center. In this period 57 of 72 individuals (health observers) adopted the use of the app. By mid-May, the app had been used by more than 20,280 users and 400 facilities and organizations. These included companies, schools, hospitals, and local governments across Japan.
Zamberg et al [34]	Utilization-focused evaluation study to identify the use of an mHealth platform for information sharing	125 members of the hospital staff	25-30 years: 28 members; 31-35 years: 24 members; 36-40 years: 18 members; 41-50 years: 29 members; 51-60 years: 24 members; >60 years: 2 members	The mHealth platform was used for 18 days from February 25, 2020, until March 13, 2020.

^a^mHealth: mobile health.

^b^ETZ: Elisabeth Twee Steden.

^c^PHR: personal health record.

^d^N/A: not applicable.

One study [24] used the corresponding mobile app (StudentLife app) as a tool for an ecological momentary assessment, aiming to assess depression and anxiety of college students to determine whether there was a change in behavior and mental health of participants in response to COVID-19.

The *number of participants* was mentioned in all selected studies. Some studies reported a relatively low numbers of participants using the corresponding app, whereas others a relatively large number of participants and users. The studies that reported the largest number of users were those described by Drew et al [26] (over 2 million users) and Menni et al [30] (2,450,569 users in the United Kingdom and 168,293 from the United States that had reported symptoms through the smartphone app). Studies that reported the lowest number of participants were the ones described by Bae et al [25] (12 patients and 24 medical staff) and Ben Hassen et al [27] (5 hospitalized patients and 5 doctors).

The *age of participants* was also reported in most of the studies except 3 [28,31,33]. The average age or age range for participants varied depending on the corresponding study.

Concerning the *follow-up duration (study duration)*, 3 out of the 12 selected studies [23,25,27], did not report the study duration. Kodali et al [28] reported the end date but did not mention the start date of app use, Drew et al [26] reported only the start date of the app launch, Ros and Neuwirth [31] reported 3 days of app use, Yamamoto et al [33] reported 14 days, Zamberg et al [34] reported 18 days, Timmers et al [32] reported 20 days, and Huckins et al [24] reported 67 days of app use. Finally, Medina et al [29] mentioned that the engagement with the app reached 32%, whereas 25% of users continued under monitoring for longer than 14 days due to persistent symptoms.

#### Outcomes

Outcome assessments were varied across studies. An overview is shown in Table 4.

**Table 4:** A comparison of outcomes for the various studies.

Paper	Primary outcomes	Positive/negative outcomes
Timmers et al [32]	The information provided by the app satisfied the user needs. Users indicated the added-value of the symptom tracker diary to be high.	Successful implementation and use of a COVID-19 app for individuals An interactive map displayed the data collected through the app. COVID-19 screening results produced at the hospital were linked to app data. Health care providers and policy makers could use the data in developing their health care strategy based on the distribution of the reported infection load.
Yamamoto et al [33]	72 health observers were identified who were in close contact with a confirmed case. Among them, 57 adopted the app, while 14 used telephone as a means for conducting investigations. Before the introduction of the app, phone interviews required more than 2 hours and four epidemiological officers for contact tracing. After the introduction of the app, only one epidemiological officer was needed to perform health observations. The visualization of health observation data improved the investigation efficiency and comprehensiveness.	The ability of individuals to record health status on a daily basis was an important countermeasure against COVID-19. The use of the app improved the efficiency and completeness of the investigation process for COVID-19 cases carried out by epidemiological officers.
Zamberg et al [34]	Three documents related to COVID-19 were made available to medical staff via the mobile platform. Information was viewed 859 times, which accounted for 35.6% of total document views. The number of sessions per day increased significantly in the study period (more than doubled) compared with the sessions per day in previous weeks. Usability evaluation: 70 (83.3%) said it was easy to find information about SARS-CoV-2. On a 10-point Likert scale, the mHealth^a^ solution scored 8.5 for time-saving and 7.6 for COVID-19 patient care assurance in daily practice.	Using the mHealth solution as a communication channel turned out to be effective within the organization for dissemination purposes during the pandemic. Daily practice was conducted by more confident and better-informed health care professionals.
Kodali et al [28]	Mixed evidence about the use of the app but mainly optimistic	Error correction, improved data collection quality, and user privacy should be considered in mHealth apps. Steps must be taken to ensure the reliability of the information provided by users. Therefore, predicting multiple verification of data entered by users could be critical.
Huckins et al [24]	With the rise of news relevant to COVID-19, college students spent more time seated, had fewer visits, and showed increases in anxiety and depression. The authors did not observe a return to baseline over the break, although they observed decreases in stress and depression that paralleled the typical drop after the final examination, suggesting some resilience in the face of COVID-19.	Mobile apps can be effectively used for tracking the mental health of college students.
Drew et al [26]	The app captures the dynamics of COVID-19 onset days before traditional measures such as positive tests, hospitalizations, or mortality. The collection emphasizes the potential usefulness of symptom monitoring in real time to help guide the allocation of resources for testing and treatment, as well as advising for tightening or loosening appropriate measures in specific areas.	With the participation of groups with underrepresented populations, the study aimed to encourage enrollment of individuals from populations that have traditionally been difficult to recruit. The study could capture correlations based on individual variations over time, a remarkable advantage over repetitive cross-sectional surveys that introduced significant variation between individuals.
Medina et al [29]	Mobile and home-based interventions were feasible for a wide range of conditions with a related risk of poor outcome from COVID-19. Approximately 10% of the patients in active monitoring presented symptoms such as shortness of breath that required escalation to a virtual provider. The median time to escalation ranged between 7 and 8 days. Patients with a pulse oximeter at home escalated a few days earlier due to reduced oxygen saturation measurements before subjective complaints of dyspnea. 2% of patients in active supervision were eventually admitted, and 3% were readmitted for persistent COVID-19 symptoms or due to complications of other underlying diseases. 9 patients monitored at home died, either due to complications related to COVID-19 or complications of another underlying disease.	Mobile engagement platforms have the potential to reduce the need for caregiver communication for patients whose symptoms are mild or persistent, freeing up the health care professionals to focus on patients who need it more.
Menni et al [30]	Besides more established symptoms such as high fever and a persistent cough, loss of smell and taste were possible prognostic factors for COVID-19. A combination of symptoms such as anosmia, fatigue, persistent cough, and loss of appetite together could identify individuals with COVID-19.	Physiological assessments of olfactory and taste function or nucleotide-based testing for SARS-CoV-2 could not be replaced by self-reporting. The authors did not know if anosmia was acquired before or after other COVID-19 symptoms, or during or after the illness.
Ross and Neuwirth [31]	This app was considered by the users as appropriate to learn and review skills relevant to COVID-19. More than 95% of respondents gave a score ≥5 for skills acquisition. 88% of respondents said it matched their health care needs. 93% of the respondents stated that the app gave them a better understanding. 87% of the respondents felt quite or very confident about the execution of the procedures, as shown in the lessons. 94% of respondents said that this particular COVID-19 training program made them feel ready to care for COVID-19 patients. 95% of respondents would suggest the application to other users.	Advantage over medical videos: It allowed the user to live the experience of seeing through a first-person view to learn through the eyes of the expert. The ability for a health care professional to instantly download locally (in a smart phone) material that can be accessed at any time in real time before, during, or after patient care interactions. When downloaded, the end user could access and view the tutorial at any time, regardless of network signal issues. Allowed health care professionals to navigate on their own or to jump to sections that were of greater importance to them
Bae et al [25]	Mobile app: usefulness showed the highest score, followed by satisfaction and perceived ease of use. Wearable vital sign monitoring perceived usefulness scored the highest, followed by perceived ease of use and satisfaction. For carers, there was an overall satisfaction score of 4.10/5.	During periods of pandemics and disasters, automated exchange of information between health care institutions plays an important role in dealing more efficiently with the problem at hand.
Ben Hassen et al [27]	Patients and doctors alike accepted the home hospitalization system very well.	Adjustments should be made for COVID-19 patients safely. Vital signs had to be measured by the patients themselves. Video communication between patients and doctors was added.
Bourdon et al [23]	Allowed doctors and patients to maintain social distance, avoiding three or four physical trips per person.	A physical appointment followed 27% of the teleconsultations. Average delay of 4.12 days between the onset of symptoms and advice, and <1 day for emergency episodes. There was 96% sensitivity and 95% specificity for the correct evaluation of the indication of a physical consultation and only 1.0% misdiagnoses.

^a^mHealth: mobile health.

In most of the cases, app users were satisfied with the educational and risk assessment information provided in the mobile apps (both citizens [32] and health personnel [31,34]). They felt reassured and informed. However, there were also concerns [28] about the measures that would need to be taken to ensure the reliability of information provided to users.

In addition, 3 studies [26,30,32] showed the feasibility of the implementation and use of COVID-19 apps to support education, self-management, and symptom tracking through diaries. Symptom tracker apps had even identified potential predictors of COVID-19, such as loss of smell and taste [30]. To this direction, mobile app sensing revealed that, during the COVID-19 pandemic, individuals were more sedentary, visited fewer locations, and showed increases in anxiety and depression [24], which is in line with the “Stay safe, stay home” policies mandated by local and national governments. However, there were concerns that cross-verification of data entered by the users could be crucial [28] and that the self-reported nature of the data collected by these apps could not replace physiological assessments and clinical examinations [30].

Additionally, mobiles apps were used for home monitoring, as reported in 4 studies [23,25,27,29], with satisfactory usability for both patients and health care professionals. These apps were focused on the management of patients who are at high risk, the moderation of exposure risk for health care workers, and the reductions of community spread through appropriate education on home-based care for individuals who are exposed or infected.

Based on the data reported in the reviewed papers, interactive maps and dashboards could be created for the quick visualization of the status summary of patients with COVID-19 and disease spread to be used by policy makers and health care providers for decision making at regional levels [26,32,33]. In addition, the dynamics of incidence days could be effectively captured, guiding allocation of resources for testing, treatment, and lockdown recommendations [26]. Mobile apps could significantly improve the efficiency and completeness of contact tracing workflows [33].

## Discussion

### Main Findings

A systematic review on COVID-19 mobile apps, as used and evaluated in research studies published in the scientific literature, is presented. Our literature search returned a significant number of records (476 unique published manuscripts), despite the short time period covered (December 2019 to June 2020), thereby showing the high interest of the scientific community in the research of mobile apps for COVID-19.

Our main finding is that, despite that the current research evidence is fragmented and requires greater methodological rigor, mobile apps have been found to benefit citizens, health professionals, and decision makers in facing the COVID-19 pandemic. In particular, mobile apps can help in solving several COVID-19–related challenges by increasing the reach of reliable information to both citizens and health professionals, decreasing misinformation and confusion, tracking symptoms and mental health of citizens, home monitoring and isolation, discovering new predictors, optimizing health care resource allocation, and reducing the burden of hospitals.

The participants in the studies were mainly young and middle-aged adults. Further studies are needed that will involve older participants, who are in greater risk of developing serious complications due to COVID-19. Understanding the needs of older individuals in the COVID-19 pandemic period would be the first necessary step toward designing and developing mobile apps to encourage their physical and mental well-being [17].

Our review, in contrast to other reviews that have not examined the evaluation of COVID-19 mobile apps in pragmatic studies [8], identified that the majority of included studies were not of high methodological quality, mainly because of their observational nature. This could be justified by the fact that the COVID-19 pandemic crisis generated an international appeal for fast response and rapid development of digital health tools by the research community, which might have inevitably led to the publication of early results by observational studies. This result can be seen as complementary to other reviews [8] that report that many of those apps are of high quality, offering many functionalities and advanced user experience. Longitudinal studies with rigorous design such as randomized controlled trials are now required to systematically assess COVID-19 mobile apps and provide strong evidence of their value. However, ethical implications might arise due to possible conflicts between liberty and privacy, equity, fairness, and justice [35-37]. In this direction, health outcomes that have scarcely been used so far, such as infection rate and quality of life, could be used as primary end points.

### Implications for Clinical Practice

The implications for clinical practice for each one of the discussed works is shown in detail in Textbox 1.

**Implications for clinical practice.**

**Timmers et al [32]**

- eHealth mobile apps for COVID-19 that support several functionalities such as tracking of symptoms and provision of accurate and timely information as well as self-assessment can be implemented in a short time to be used by individuals.
- Those apps provide valuable information to both governments and health care providers, since they support monitoring of patient health status and provide summary statistics regarding the progress of health.
- Such apps could be used in future outbreaks of other viruses to support all involved stakeholders.

**Yamamoto et al [33]**

- Such apps could be used in future outbreaks of other viruses to support all involved stakeholders.

**Zamberg et al [34]**

- Mobile health (mHealth) apps could help solve some of the COVID-19 challenges by providing more accurate and timely information to health care professionals. This benefit is sourced by the centralized management and storage of main, up-to-date, validated, and easily accessed information in one platform.
- mHealth apps used in health care organizations as communication tools of validated information should be assessed in the context of clinical studies, with regards to their impact on clinical outcomes.

**Kodali et al [28]**

- According to users, the app should be enhanced with additional functionalities including tracking of location, provision of up-to-date information on COVID-19 as well as information on areas with high/medium/low epidemiological burden, and deployment in nonmobile platforms.

**Huckins et al [24]**

- According to the findings, since the beginning of the COVID-19 pandemic, there has been an extended negative impact both on the physical health of individuals, resulting in an increased number of deaths, and in their mental health, resulting in changes in their behavior.

**Drew et al [26]**

- The research of a broader range of potential risk factors for COVID-19 results will be largely supported by the use of the app within several large epidemiology cohorts for which there are a large amount of data on lifestyle, diet and health factors, and genetic information.
- It will be useful to deploy the tool in several clinical studies, in centralized actions related to biobanking, and in health care worker monitoring programs.

**Medina et al [29]**

- The intervention impacts several organizational matters and provides answers regarding the following questions related to COVID-19:
  Which situations should we focus on to provide effective care to prevent patient admission to intensive care unit and mechanical support?
  How can those situations be better supported by increased patient self-reporting?
  Can we predict, based on time data, future inpatient demand?
  How can we manage not having conflicts between patient choice and availability of treatment?

**Menni et al [30]**

- Routine screening for COVID-19 could also include loss of sense of taste and smell. These two symptoms should also be included in the related symptom list provided by the World Health Organization.

**Ros and Neuwirth [31]**

- The implementation and deployment of the described digital tutorials, as an effective and swift global public health educational tool, help alleviate the burden that hospitals, health care professionals/responders, and patients face due to the COVID-19 pandemic.

**Bae et al [25]**

- Episode triage, timely diagnosis, isolation of patients, and their treatment can be largely supported by telemedicine solutions.

**Ben Hassen et al [27]**

- Mobile apps can be used by patients, their caregivers, and health care professionals to better monitor and manage patient health status in the context of patient hospitalization. Moreover, such apps are cost-effective, reliable, and safe, providing important economic benefits to hospitals. Overall, they are accepted to a great extent by patients and individuals.

**Bourdon et al [23]**

- The use of telemedicine tools for consultation purposes improved the access to health care services for patients with ophthalmological problems. At the same time, such tools preserved social distancing and sanitary measures.
- Such tools can be used in emergency situations by ophthalmological patients who have limited access to specialized care.

As reported in the textbox, eHealth solutions can be implemented rapidly and can offer essential tools in supporting the COVID-19 pandemic for all stakeholders including citizens, health care providers, policy makers, and governments. The impact of the COVID-19 pandemic goes beyond the illness and deaths that are directly associated with the SARS-CoV-2 pathogen and toward an expanded scope encompassing mental health and several behavioral changes.

However, there are still several areas to explore, such as better geolocation tracking; timely COVID-19 updates; deployment on nonsmartphone platforms; better incidence visualization and prediction; investigation of a much broader range of putative risk factors for COVID-19 outcomes (based on other longitudinal data on lifestyle, diet, genetics, etc); and the integration with clinical systems, digital health literacy, and engagement with mobile apps.

Apps such as the ones presented in these studies can give the research community the opportunity to monitor long term effects of COVID-19; answer questions such as the number of people truly affected, why some people get sicker than others, and how long people can stay immune to the disease; and perform better resource allocation of medical equipment.

In case of future disease outbreaks, the mobile apps already developed for COVID-19 can be valuable tools ready to support people, health care providers, and policy makers. This study can provide a guide for future developers and researchers regarding the current methodological gaps and challenges that need to be addressed to develop well-designed and evaluated apps for similar future circumstances.

### Limitations

We used terms related to mobile apps, mHealth, and mobile phones for our literature search according to the review objectives and did not use related terms such as “telehealth,” which might have resulted in an inadvertent omission of studies. Our literature search was conducted in a limited number of bibliographic databases (Pubmed, Scopus, and the WHO’s COVID-19 database), which nevertheless have been largely used worldwide. The gray literature was not explored. The interrater reliability between the authors was not assessed. A meta-analysis was not possible due to the heterogeneity of the included studies. For many cases, the quality of the study might not reflect the quality or the effectiveness of the developed mobile app. As such, useful and effective apps might have not been included in this review study due to the limited quality of the related studies.

## Appendix

Multimedia Appendix 1 PRISMA (Preferred Reporting Items for Systematic Reviews and Meta-Analyses) 2009 checklist.

## Abbreviations

EPHPP: Effective Public Health Practice Project
mHealth: mobile health
PRISMA: Preferred Reporting Items for Systematic Reviews and Meta-Analyses
WHO: World Health Organization
